# Interleukin-26 (IL-26) is a novel anti-microbial peptide produced by T cells in response to staphylococcal enterotoxin

**DOI:** 10.18632/oncotarget.24603

**Published:** 2018-04-13

**Authors:** Anders Woetmann, Morten Alhede, Sally Dabelsteen, Thomas Bjarnsholt, Morten Rybtke, Claudia Nastasi, Thorbjørn Krejsgaard, Mads Hald Andersen, Charlotte M. Bonefeld, Carsten Geisler, Michael Givskov, Niels Odum

**Affiliations:** ^1^ Department of Immunology and Microbiology, University of Copenhagen, Copenhagen, Denmark; ^2^ Department of Clinical Microbiology, Rigshospitalet, Copenhagen, Denmark; ^3^ Department of Odontology, University of Copenhagen, Copenhagen, Denmark; ^4^ Costerton Biofilm Center, University of Copenhagen, Copenhagen, Denmark; ^5^ Center for Cancer Immune Therapy (CCIT), Department of Hematology, Copenhagen University Hospital, Herlev, Denmark; ^6^ Singapore Centre for Environmental Life Sciences Engineering (SCELSE), Nanyang Technological University, Singapore

**Keywords:** IL-26, antimicrobial peptide, staphylococcus aureus enterotoxins, chronic wounds, superantigens staphylococcus pseudomonas, Immunology

## Abstract

Anti-microbial peptides are produced at outer and inner surfaces by epithelia and innate immune cells in response to bacterial infection. *Staphylococcus aureus* is an enterotoxin producing, Gram-positive pathogen, which is a major cause of soft tissue infections and life-threatening bacteremia and sepsis. Here we show that (i) skin T cells in chronic wounds infected with *S. aureus* express interleukin-26 (IL-26) *in situ*, (ii) staphylococcal enterotoxins (SE) trigger IL-26 expression in T cell lines and primary skin T cells, and (iii) IL-26 triggers death and inhibits biofilm formation and growth of *S. aureus*. Thus, we provide novel evidence that IL-26 is an anti-microbial peptide produced by T cells in response to SE. Accordingly, we propose that IL-26 producing T cells take part in the innate immune response to SE producing *S. aureus* and thus play a novel role in the primary innate immune defense in addition to their classical role in adaptive immunity.

## INTRODUCTION

*Staphylococcus aureus* is a major cause of life-threatening infections such as bacteremia, sepsis, and endocarditis and account for approximately 19,000 deaths per year in the United States [1]. *S. aureus* can grow in biofilms and produces an array of proteins interfering directly- and in-directly with host immune responses and antibiotic therapy [2, 3]. These factors include staphylococcal protein A, staphylococcal binder of immunoglobulin, and a large family of staphylococcal enterotoxins (SE) (reviewed in 3). SE are known as super-antigens because they directly cross-link MHC class II on antigen-presenting cells (APC) and the T cell receptor (TCR) on T cells (expressing the appropriate TCR- Vβ chain) without prior antigen-processing by the APC [4–6]. Thus, SE are able to elicit an aberrant immune response [4] while at the same time able to block specific T cell receptor and cytokine responses [7, 8]. Inversely, multiple host defense mechanisms against *S. aureus* have been identified including anti-microbial peptides, antibodies, neutrophils and IL-17 producing helper T (TH17) cells [9]. Anti-microbial peptides are produced at outer and inner surfaces by epithelia and innate immune cells in response to bacterial infection. Host responses to *S. aureus* involve cathelicidin peptides like LL-37 as well as α- and β-defensins [10, 11]. SE are among the most potent activators of T cells and as little as a few SE molecules are sufficient to trigger T cell activation [4] indicating that T cells may play a unique role by sensing staphylococcal enterotoxins at extremely low concentrations [4]. In particular, TH17 cells are believed to be important in the host defense against *S. aureus*, partly through recruitment of neutrophils and partly through the production of the cytokines IL-17 and IL-22, which in turn stimulate the production of anti-bacterial substances including LL-37 and defensins [10–14]. IL-26 is a newly described cytokine belonging to the IL-10 super-family [15], which has recently been implicated in autoimmune diseases such as rheumatoid arthritis, psoriasis, and colitis [16, 17]. However, the biological function of IL-26 is far from understood. Recently, IL-26 was shown to possess anti-bacterial activity against a wide range of bacteria including *S. aureus* [18] suggesting a broader role in host defenses against bacteria [19]. Here we show that SE triggers IL-26 expression in T cells and that IL-26 inhibits *S. aureus* growth, survival and biofilm formation indicating that T cells sense and respond to enterotoxin-producing *S*. *aureus* by expression of the newly described anti-microbial cytokine, IL-26.

## RESULTS AND DISCUSSION

**Figure 1:**
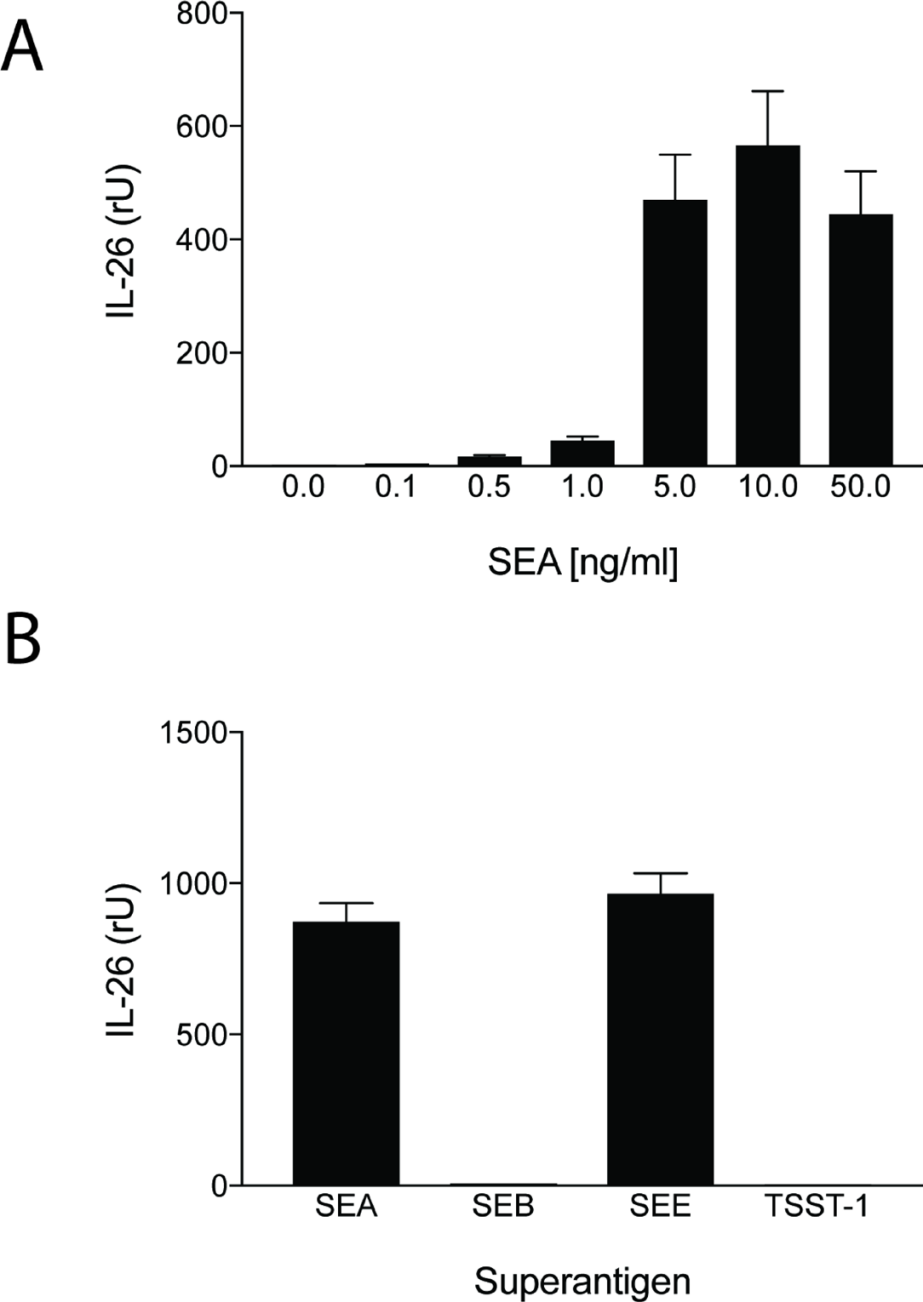
Staphylococcal enterotoxin triggers IL-26 expression in antigen specific CD4+ TH22 T cells SEA- and SEE- responsive CD4+ human TH22 T cell lines (22 and data not shown) were cultured in a humidified atmosphere at 37 degrees Celsius and stimulated with increasing concentrations of SEA prior to analysis for IL-26 expression by RT-PCR (34) (**A**); or stimulated with or without recombinant SEA, SEB, SEE, and toxic shock syndrome toxoid (TSST) at 10 ng/ml for 24 hours prior analysis for IL-26 expression by RT-PCR (**B**).

**Figure 2:**
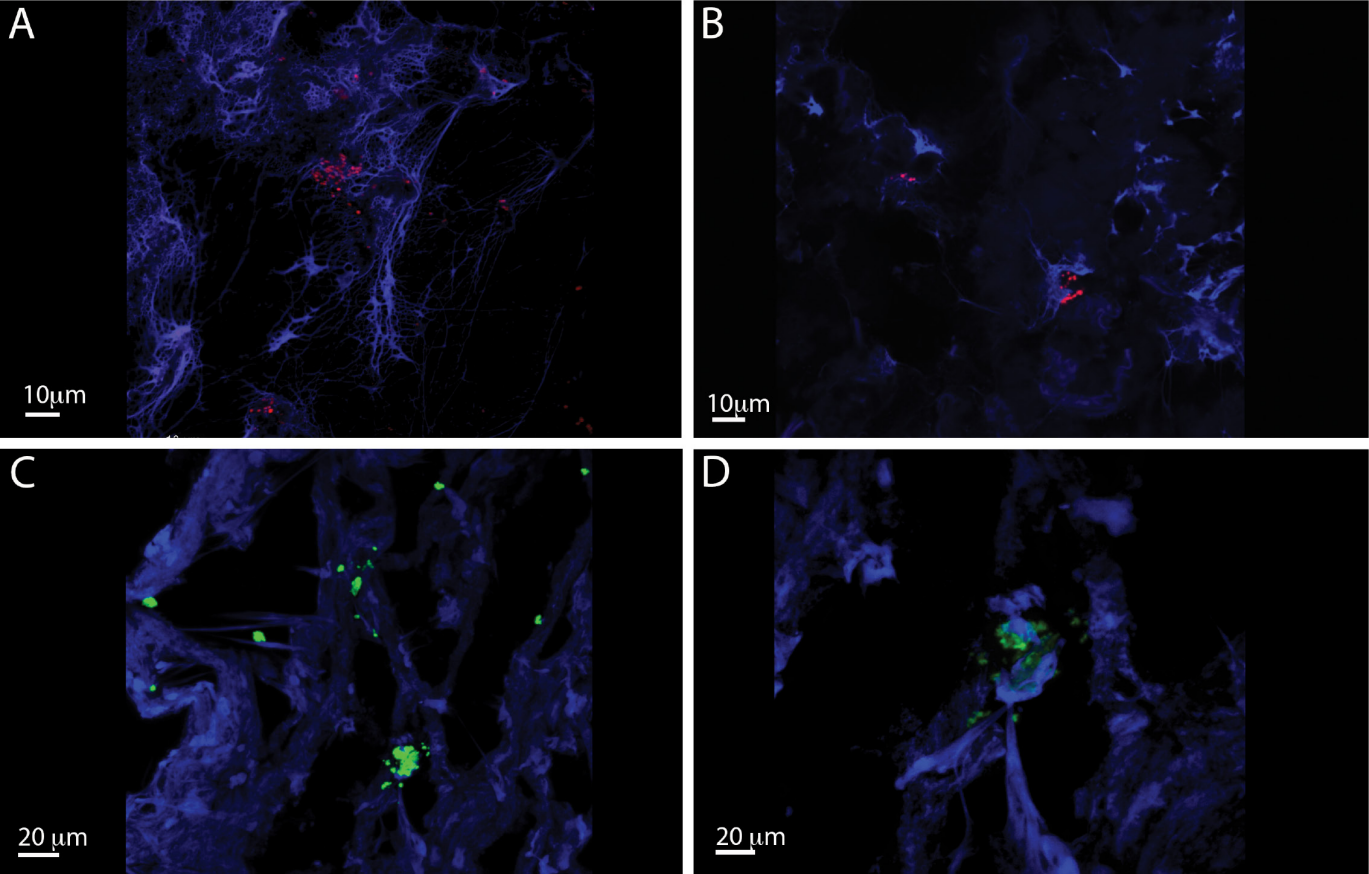
IL-26 expression is Staphylococcus aureus infected chronic venous wounds Representative CLSM images of *S. aureus* (**A** and **B**) and IL-26 (**C** and **D**) in chronic wounds. The bacteria were detected by PNA-FISH with an TxR-labeled *S. aureus*-specific probe (red) (bars indicate 10 μm) and FITC labeled il-26 specific antibody (green) (bars indicate 20 μm). DAPI was used as blue counterstain (host cells).

**Figure 3:**
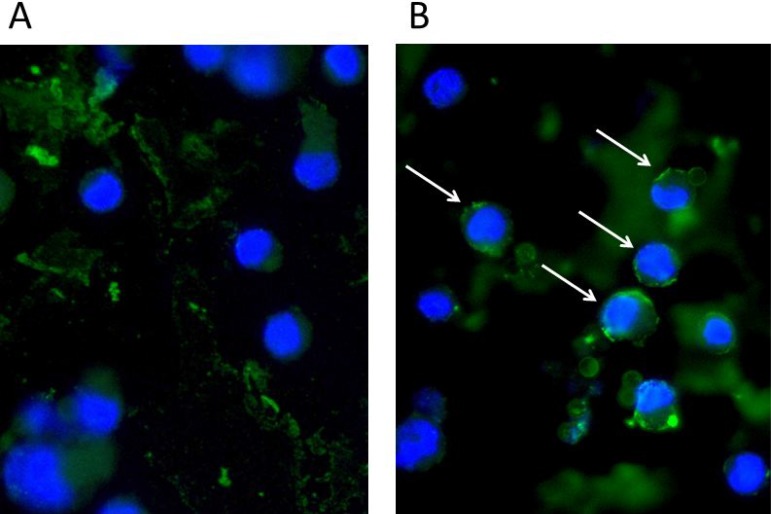
Staphylococcal enterotoxin (SEA) triggers IL-26 expression in responsive primary skin T cells Normal human skin-derived primary T-cells were stimulated for 24 h without (**A**) or with SEA (100 ng/ml) (**B**) and stained with IL26 (green). Nuclei stained with DAPI (blue). A subpopulation of T-cells produces IL26 when stimulated with SEA (arrows).

IL-26 has been implicated in chronic inflammation and autoimmunity whereas its role in infectious diseases is unclear. However, recent data indicated that IL-26 is an anti-microbial peptide that kills extracellular bacteria such as *S. aureus* [18]. As *S. aureus* produce toxins that are extremely potent stimulators of T cells [4], we hypothesized that T cells may play a role in the early antimicrobial response to bacterial toxins by producing IL-26, which, in turn, inhibits bacterial growth and immune evasion. Accordingly, we examined whether staphylococcal toxins induced IL-26 expression in human T cells. As shown in Figure 1, staphylococcal enterotoxin-A (SEA) induced IL-26 expression in a concentration-dependent manner in human SEA-sensitive CD4+ T cell lines [20]. Thus, IL-26 mRNA was induced at SEA concentrations as low as 1-5 ng/ml, whereas optimal IL-26 induction was observed at a SEA concentration of 10 ng/ml (Figure 1A). Our observation that IL-26 induction was not further increased with higher concentrations of SEA (> 10 ng/ml) was in keeping with previous findings that SEA at high concentrations triggers apoptosis in CD4+ T cells [4]. The IL-26 response was highly specific for SEA and the closely related enterotoxin SEE (Figure 1B). In contrast, enterotoxins such as SEB and TSST did not trigger an IL-26 response in SEA/SEE- sensitive T cell lines (Figure 1B). Reversely, SEA and SEE did not induce IL-26 expression in T cell lines (data not shown), which did not express SEA/SEE- responsive TCR Vβ chains [20]. To address whether clinical infections with *S. aureus* were associated with expression of IL-26 *in situ*, we examined for IL-26 expression in skin wounds chronically infected with *S. aureus*. Accordingly, we used a Texas Red (TxR)-labelled *S. aureus* specific probe in a FISH assay [21, 22] and a FITC- labeled IL-26 specific antibody to examine IL-26 expression in tissue sections. Staining for *S. aureus* (Figure 2A, 2B, red stain) and co-staining for IL-26 (Figure 2C, 2D) showed the presence of *S. aureus* and expression of IL-26 in the same wounds (Figure 2). Accordingly, we addressed whether purified SEA induced IL-26 in skin-resident T cells from healthy individuals. To this end, primary skin T cells isolated from healthy skin specimens were stimulated *ex vivo* with SEA for 24 hours prior to cyto-histochemical analysis for IL-26 expression using the FITC IL-26 conjugated antibody. As shown in Figure 3, IL-26 was expressed in a fraction of SEA responsive skin T cells following SEA exposure *ex vivo* (Figure 3B versus Figure 3A; green labeling as indicated by arrows). In contrast, IL-26 was not expressed *ex vivo* in SEA- non-responsive skin T cells from healthy, uninfected individuals (Figure 3A and data not shown).

**Figure 4:**
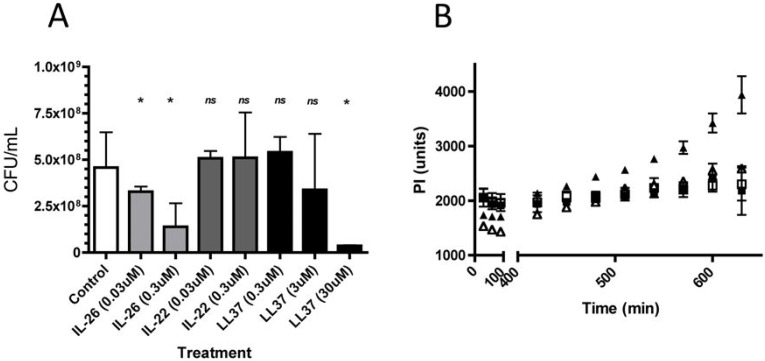
IL-26 inhibits growth and triggered death in cultures of *S. aureus* *S. aureus* were grown for 24 hours in the presence or absence of varying concentrations of IL-26, IL-22, and LL37. (**A**) The number of colony forming units was measured by spread-plating in LB agar following exposure to IL-26 (left), IL-22 (middle), and LL37 (right) and (**B**) cell death was measured following culture without (open triangles) or with IL-26 (0.3 uM) (closed triangles) as fluorescence intensity following propidium iodide uptake as described elsewhere [22, 23].

The outer walls of bacteria such as *S. aureus* are negatively charged and anti-microbial peptides like LL-37 are characterized by an ability to bind to negatively charged membranes [11]. As IL-26 is a polycationic protein with a strong positive charge and anti-microbial properties [18, 23], we addressed whether IL-26 functions as an anti-bacterial peptide similar to LL-37. Accordingly, *S. aureus* were incubated in growth medium for 24 hours in the presence or absence of IL-26 and the number of colony forming units was measured by spread-plating in LB agar as described elsewhere [21, 22]. As shown in Figure 4A, IL-26 significantly reduced the number of colony forming units in a concentration dependent manner. At a concentration of 0.3 μM, IL-26 reduced the number of colony forming units (CFUs) to 50% (*p* < 0.05, Figure 4A, third column from the left). IL-22 and IL-26 are related cytokines belonging to the IL-10 superfamily and expressed in tandem by some TH17 and TH22 cells [16, 17]. Accordingly, we addressed whether IL-22 also inhibited *S. aureus* cultures *in vitro*. However, as shown in Figure 4A (middle rows), IL-22 had no effect on the number of CFUs indicating that the anti-bacterial effect was specific for IL-26. Interesting, IL-22 does not possess the physical/chemical characteristics of IL-26 (such as a predicted basic isoelectric point above 24) and lacks its ability to bind to negatively charged membranes suggesting that these features play a key role of IL-26 to function as an anti-bacterial peptide [18]. As mentioned above, LL-37 also binds to negatively charged membranes and as expected, LL-37 profoundly inhibited growth of *S. aureus* cultures (Figure 4A, right columns). Notably, 10 times higher concentrations of LL-37 were required to inhibit *S. aureus* (Figure 4A) indicating higher sensitivity to IL-26 than to LL-37. To address whether IL-26 bactericidal, we measured DNA release by fluorescent propidium iodide (PI) staining [22]. As shown in Figure 4B, 24 hours of incubation with IL-26 induced a significant increase in free DNA/PI in stationary bacterial cultures (Figure 4B, closed triangles), when compared to vehicle (Figure 4B, open triangles) indicating that IL-26 induced a time-dependent increase in bacterial death.

**Figure 5:**
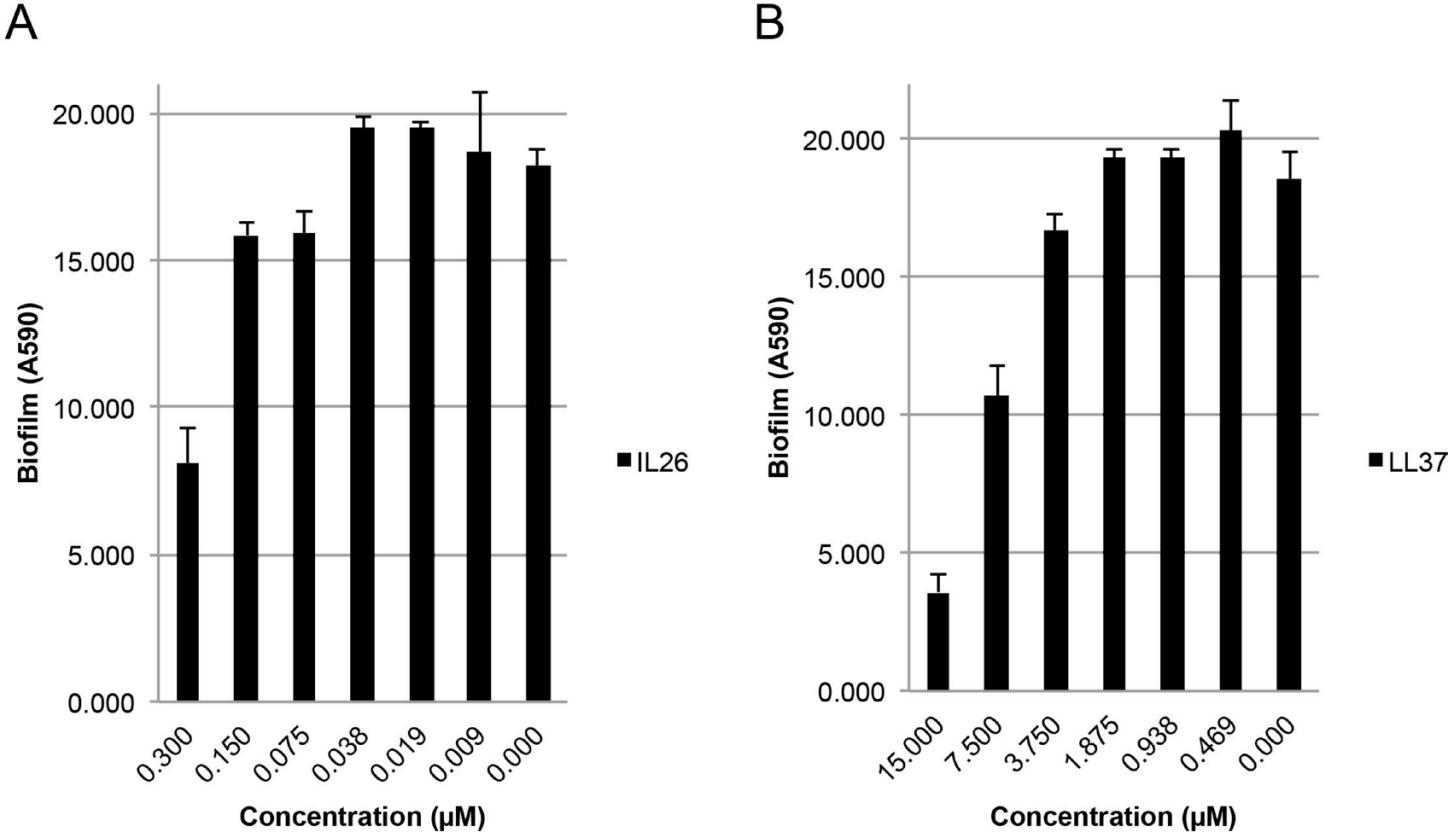
IL-26 inhibits formation of biofilms in cultures of *S. aureus* *S. aureus* biofilm formation in cultures treated without or (**A**) with IL-26 at varying concentrations and (**B**) LL37 at varying concentrations for 24 hrs prior to biofilm quantification with crystal violet as described in materials and methods.

It has become increasingly acknowledged that biofilm formation by aggregating bacteria comprises a serious clinical problem [24]. Thus, generation of a biofilm creates a barrier, which shields the bacteria from antibiotics and the immune system [24]. Because LL-37 inhibits biofilm formation in a wide array of bacteria including *S. aureus* [10, 25], we investigated whether IL-26 had a similar effect on biofilm formation by *S. aureus in vitro*. As measured by crystal violet staining, biofilm formation was profoundly inhibited by IL-26 in a concentration-dependent manner (Figure 5A). Thus, crystal violet staining (amount of attached biofilm) was decreased by more than 50% by IL-26 at a concentration of 0.3 μM (Figure 5A, right). Even at IL-26 concentrations as low as 75 nM, a reduction in the amount of biofilm was observed (Figure 5A). As expected, LL-37 also inhibited biofilm formation by *S. aureus* (Figure 5B). Interestingly, IL-26 appeared to be a more potent inhibitor of biofilm formation than LL-37 (Figure 5A versus Figure 5B), which is in line with the observation above that colony formation by *S. aureus* was more sensitive to IL-26 than to LL-37. Since the reduction in biofilm is paralleled by a decrease in bacterial viability (Figure 4B), it is most likely that the inhibition of biofilm formation is caused by the antibacterial activity of IL-26.

Given the biophysical/chemical characteristics of IL-26 [16], it might be expected that the anti-bacterial capacity of IL-26 is not limited to *S. aureus*. Indeed, Meller *et al.* [18] reported that IL-26 inhibited growth of other bacteria including *Pseudomonas aeruginosa*. In accordance, we observed that IL-26 inhibited colony and biofilm formation by *P. aeruginosa* with a similar potency as was observed for *S. aureus* inhibition (Supplementary Figure 1). Accordingly, our data confirm [18] and extend the hypothesis that IL-26 inhibits a wider spectrum of bacteria and thus, has a general role as an anti-bacterial peptide.

It is well known that infection with *S. aureus* plays a pathogenic role in disorders like impetigo, ecthyma, sepsis, and chronic wounds. However, it has become clear that colonization with SE-producing *S. aureus* may also drive deregulation of signal transducers and activators of transcription (STAT), cytokine release, and/or disease progression of chronic skin diseases such as atopic dermatitis and cutaneous T cell lymphoma [26–30]. Interestingly, clinically infections with *S. aureus* and other bacteria generally occur after diagnosis [31], i.e. after skin lesions with compromised skin barriers have become evident supporting the notion that SE producing bacteria may aggravate CTCL without necessarily playing a primary etiological role triggering the disease in the first place [31]. As IL-26 expression is greatly enhanced in CTCL lesions [32], it may be speculated that an increased expression of IL-26 *in situ* reflects an increased burden of SE-producing *S. aureus* in CTCL skin lesions as previously reported by Jackow *et al.* [33]. Furthermore, SE have been proposed to play a role in autoimmune and chronic inflammatory diseases such as rheumatoid arthritis and Crohn's disease [34–36]. As a series of recent studies implicated IL-26 as a potential key player in autoimmunity [37], we propose that IL-26 - in addition a putative intrinsic role in autoimmunity - may also be a marker of the involvement of bacterial superantigens in some cases of autoimmunity and chronic inflammation.

In conclusion, the present study provides the first evidence that IL-26 functions as an antibacterial peptide in response to T cell exposure to SE. This finding makes sense since IL-26 is produced by specific T cell subsets (TH-17 and TH-22), which play a key role in antibacterial responses by attracting and activating neutrophils and other innate immune cells through release of IL-17 family cytokines, chemokines, and other mediators [12]. As staphylococcal enterotoxins bind with very high affinity to T cells expressing the appropriate TCR-Vβ, these T cells can respond to enterotoxins are extremely low concentrations [4]. Importantly, previously activated human CD4 T cells express MHC class II molecules that are high-affinity receptors for staphylococcal enterotoxins [4, 20]. Notably, MHC class II ligation triggers a series of signaling events in human T cells involving PLCγ activation, enhanced cytokine expression, and augmented growth of CD4 T cells [38–42]. Moreover, simultaneous crosslinking of MHC class II and TCR act in synergy to trigger signal transduction in T cells [43] suggesting that previously activated CD4^+^ T cells expressing the relevant TCR-Vβ chains and MHC class II molecules are particularly prone to respond to low concentrations of staphylococcal enterotoxins. Because enterotoxin recognition is also independent of prior antigen processing by APCs and T cell priming, we propose that IL-26 producing T cells (in addition to their role in adaptive immunity) play a direct role in innate immunity against bacteria such as enterotoxin producing staphylococci.

## MATERIALS AND METHODS

### Cells

Staphylococcal enterotoxin (SE) responsive human CD4 TH22 (IL-22 positive, IL-17- negative) T cell lines and clones were specific for MHC class II alloantigens and SEA and SEE as described elsewhere (22,42, and unpublished data). Primary, skin-resident T cells were isolated from skin specimens as described [44]. T cells were incubated with or without SE for varying periods of times in RPMI-1640 supplemented with 2 mM L-glutamine, 100 mgml–1 penicillin/streptomycin (all from Sigma-Aldrich), 10% pooled human serum (Blood Bank, State University Hospital, Copenhagen, Denmark) in a humidified atmosphere at 370 as described [45–46].

### Chronic venous leg ulcers

4-mm punch biopsy specimens from chronic venous leg ulcers were obtained with the acceptance of the patients and in accordance with biomedical project protocols H-B-2008-023 and KA-20051011, which were approved by the Danish Scientific Ethical Board. Wound biopsy material was collected from 8 patients by a surgical team before cleansing and surgical preparation of the wound. The material was immediately frozen to -80 degrees.

### RNA isolation and reverse transcriptase-PCR

Total RNA was isolated using RNeasy Mini Kit (Qiagen, Ballerup, Denmark) according to the manufacturer's instructions and reverse transcriptase-PCR was performed as described elsewhere [47, 48] (all reagents were from Invitrogen, Paisley, UK; except Taq polymerase, which was from New England Biolabs, Danvers, MA, USA). Primers were designed with Primer3 v 0.4.0 software (Duke-NUS Graduate Medical School, Singapore) and synthesized by Eurofins MWG GmbH (Ebersberg, Germany). Primer sequences for IL-26 amplification, F: ATTGCAAGGCTGCAAGAAAA R: TC CAGTTCACTGATGGCTTTG, primer sequences for GAPDH amplification, F: CCATGGAGAAGGCTGGGG R: CAAAGTTGTCATGGATGACC.

### PNA-FISH

The tissue sections were analyzed by FISH with PNA probes as described elsewhere [49, 50]. The PNA probe in hybridization solution (AdvanDx, Inc., Woburn, MA) was added dropwise to each tissue section, which was then covered with a coverslip and hybridized in a PNA-FISH workstation (AdvanDx, Inc.), which was covered with a lid, at 55°C for 90 min. PNA probe solutions were used: a Texas Red (TxR)-labeled *S. aureus*-specific probe. The slides with tissue sections were washed in a wash solution (AdvanDx, Inc.) at 55°C for 30 min, air dried, mounted with Vectashield mounting medium with 4′,6′-diamidino-2-phenylindole (DAPI; Vector Laboratories), and covered with a coverslip [49]. The tissue sections were examined as described below.

### Immunofluorescence

For co-staining with bacteria. Frozen sections with labeled bacteria were incubated o.n. with mouse monoclonal anti-human IL26 (R&D systems, UK, MAB13751) diluted 1:40 in PBS with 2,5% BSA. Detection with secondary Alexa 488, rabbit anti-mouse antibody (Alexa fluorocrome, Thermo Scientific, USA), and mounted with prolong Gold antifade (Vector Laboratories). The tissue sections were examined as described below.

### Bacterial strains

*Staphylococcus aureus* strain 8324 and *Pseudomonas aeruginosa* strain PAO1 were grown in TSB media supplemented with 1% glucose at 37°C as described [49, 50].

### Bacterial culture

In a 96-well plate (BD Falcon 353072), 1 × 10^5^ CFU per well bacteria were incubated with different peptide concentrations (in serial dilutions of 1:10 across the plate) in a solution of buffer containing sterile 10 mM sodium phosphate (pH 7.4) and incubated for 24 h at 37°C. Negative control wells contained bacteria with no peptide. Serial dilutions were then carried out in sterile 1x PBS (Fisher Scientific) (pH 7) and plated in triplicate on LB Agar plates, incubated (37°C, 24 h) and counted.

### Biofilm formation assay

Biofilm attachment assays were performed in a 96-well microtiter plate (BD Falcon 353072), as previously described. Overnight cultures of *S. aureus* were grown ON in TSB. The ON cultures were diluted to an optical density (600 nm) of ∼0.05 in TSB + 1% glucose and the desired concentration of peptide or cytokine. 200 μl culture was added to the wells. The plates were incubated (24 h, 37°C) for *S. aureus* to grow and adhere to the wells. After incubation, the medium was discarded, and plates were gently washed three times with 200 μl sterile phosphate buffered saline (PBS). Thereafter, plates were air dried and stained with 50 μl crystal violet (CV; 0.1%) for 15 min. Excess stain was decanted off and, plates were washed three times with sterile distilled water. The biofilms were dissolved in 200 μl of 95% ethanol and the OD590 nm was measured in an automatic spectrophotometer. To compensate for background absorbance, values from the sterile medium and CV were averaged and subtracted [51, 52].

### Image acquisition and analysis

Microscopic observations of the tissue sections were performed with a Zeiss Imager. Z2 microscope with LSM 710 CLSM and the accompanying software Zeiss Zen 2010 v. 6.0. (Zeiss, Germany) equipped with an argon laser and a helium-neon laser for excitation of the fluorophores. Multichannel simulated fluorescence projection images were generated by using the IMARIS software package (Bitplane AG, Zurich, Switzerland).

## SUPPLEMENTARY MATERIALS FIGURES AND TABLES


