# Supplementary material for: Interleukin-26 (IL-26) is a novel anti-microbial peptide produced by T cells in response to staphylococcal enterotoxin
Source: Oncotarget. 2018 Apr 13;9(28):19481–9. doi: 10.18632/oncotarget.24603 (PMC5929403; doi:10.18632/oncotarget.24603)
Supplement: Supplementary file 1 [file oncotarget-09-19481-s001.pdf]

Interleukin-26 (IL-26) is a novel anti-microbial peptide produced by T cells in response to staphylococcal enterotoxin

SUPPLEMENTARY MATERIALS

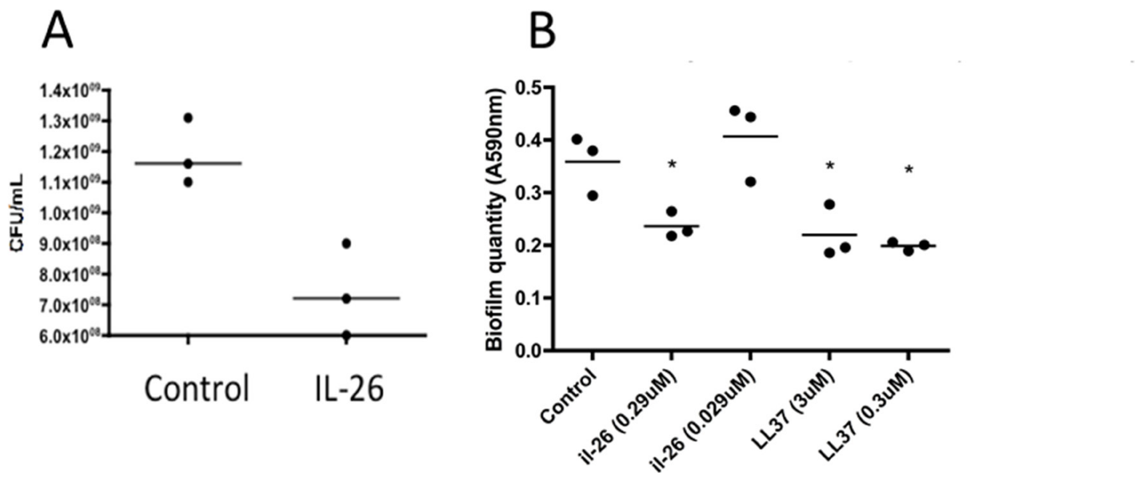

Supplementary Figure 1: IL-26 inhibits growth and biofilm formation in cultures of *P. aeruginosa.* (**A**) *P. aeruginosa* were grown for 24 hours with and without IL-26 and the number of colony forming units was measured by spread-plating in LB agar as described above and (**B**) *P. aeruginosa* biofilm formation in cultures treated without or with IL-26 at varying concentrations and LL37 at varying concentrations for 24 hrs prior to biofilm quantification with crystal violet as described in materials and methods.
